# Biological Features and Population Growth of Two Southeastern European *Tribolium confusum* Jacquelin du Val (Coleoptera: Tenebrionidae) Strains

**DOI:** 10.3390/insects11040218

**Published:** 2020-04-02

**Authors:** Nickolas G. Kavallieratos, Goran Andrić, Marijana Pražić Golić, Erifili P. Nika, Anna Skourti, Petar Kljajić, Nikos E. Papanikolaou

**Affiliations:** 1 Laboratory of Agricultural Zoology and Entomology, Department of Crop Science, Agricultural University of Athens, 75 Iera Odos str., 11855 Athens, Attica, Greece; nick_kaval@aua.gr (N.G.K.); goran.andric@pesting.org.rs (G.A.); marijana.prazic@pesting.org.rs (M.P.G.); erifilinika@aua.gr (E.P.N.); annaskourti@aua.gr (A.S.); 2 Institute of Pesticides and Environmental Protection, Banatska 31b, 11080 Belgrade, Serbia; petar.kljajic@pesting.org.rs; 3 Directorate of Plant Produce Protection, Greek Ministry of Rural Development and Food, 150 Sygrou Ave., 17671 Athens, Attica, Greece

**Keywords:** confused flour beetle, strain, barley, white rice, demography

## Abstract

A study of the biological features and the potential population growth between two laboratory strains of the confused flour beetle, *Tribolium confusum* Jacquelin du Val (Coleoptera: Tenebrionidae) from Greece and Serbia is conducted on cracked barley and cracked white rice. The results show that, at a species level, *T. confusum* is able to complete development on cracked barley but not on cracked white rice. Therefore, cracked white rice proves to be an unsuitable commodity for *T. confusum*. Larval development on cracked barley is significantly shorter for the Serbian compared to the Greek strain (37.7 and 49.7 days, respectively), but pupal development does not differ between the two strains (6.2 days for both strains). Additionally, male longevity does not differ between the Greek and Serbian strains (144.4 and 151.4 days, respectively), while female longevity is significantly shorter for the Serbian (151.7 days) compared to the Greek strain (186.6 days). Fecundity does not differ between the two strains (11.3 and 17.7 eggs/female for the Greek and the Serbian strain, respectively), whilst survival is higher for the Serbian strain on both tested commodities. The values of the net reproductive rate, the intrinsic rate of increase and the finite rate of increase on cracked barley are significantly higher for the Serbian (7.27 females/female, 0.025 female/female/day and 1.026, respectively) compared to the Greek strain (2.91 females/female, 0.014 females/female/day and 1.014, respectively). It therefore is expected that different strains of *T. confusum* may exhibit variable phenology as well as potential population growth. Additionally, we expect our results to have bearing on the management of this species.

## 1. Introduction

The confused flour beetle, *Tribolium confusum* Jacquelin du Val (Coleoptera: Tenebrionidae) is a long-lived species that can seriously and rapidly infest stored-products [1,2,3]. It infests cereal grains or their products and is usually found in mills, bakeries, warehouses and pet stores [4,5]. It is regarded as a secondary colonizer since it cannot easily develop in sound grain kernels [6,7]. It can also damage dried plants or fruits, dairy products, oilseeds, nuts, animal feed, cotton and spices [8,9]. Adults are approximately 3.5 mm long and have a reddish brown color, while larvae can reach a 6–7 mm length [9]. Adults have fully developed wings, but there is no record that they fly [10,11]. *Tribolium confusum* is widespread over the globe because it is able to breed in temperatures from 19 to 37.5 °C and survive at a low relative humidity (>1%) [4]. Due to the fact that *T. confusum* is globally spread and tolerant to several insecticides [12,13,14,15], its economic importance is considered high [2]. Potentially, it has an important impact on public health since it produces defensive secretions that cause skin irritation through severe itching, and it also can cause respiratory disorders [16].

Different strains often exhibit contrasting biological traits and genetic variability because they have been geographically isolated, exposed to different selection pressures, including insecticides [17], and/or adapted to various local environments [18,19,20]. Different strains of the red flour beetle, *Tribolium castaneum* (Herbst) (Coleoptera: Tenebrionidae), the rice weevil, *Sitophilus oryzae* (L.) (Coleoptera: Curculionidae) and *T. confusum* have different behavioral responses to kairomones and pheromones [20], mating and lateralized traits [21] or developmental time and fecundity [22], respectively. Furthermore, susceptibility to grain protectants also is variable within strains of the same stored-product insect species [23,24,25,26]. Nevertheless, the origin of strains of some stored-product insects had insignificant or a non-significant effect on several life history traits [27,28,29]. 

Biological features of insects, such as development, survival, longevity and fecundity are, in turn, critical aspects of their life history [30,31,32,33]. The knowledge of these parameters could be useful for the prediction of insect phenology [22]. Moreover, the tabulating of birth and death rates results in the construction of life tables which constitute a powerful demographic technique that provides a comprehensive and detailed description of the development, survivorship and reproduction of insect populations. The calculation of several demographic parameters indicates the insects’ performance and reveals the optimal time of suppression of their densities [32,34,35]. The values of the finite rate of increase, intrinsic rate of increase, net reproductive rate, mean generation time and doubling time are important demographic parameters that indicate species population growth [33,35,36,37].

Different mathematical models and statistical techniques have been applied for the interpretation of the population outcome of stored-product insects [38,39,40,41,42,43]. Several studies have shown that feeding on different types of commodities can affect the demography of stored-product insect pests such as the life history traits, the survival or duration of larvae and the intrinsic rate of increase [33,35,44,45,46,47,48,49]. Although *T. confusum* is a severe stored-product insect, there is limited knowledge, which mostly comes from the seventies and eighties, on the demography of this species. Hardman [50,51], for example, incorporated values of life table parameters (e.g., duration of egg, larval and pupal development, mortality of eggs, larvae and pupae, sex ratio, fecundity) recorded under constant temperatures in wheat flour into deterministic and stochastic models to predict the population growth of *T. confusum*. Later, Daly and Ryan [52], by studying three population densities of *T. confusum* in wheat flour, found that mortalities recorded at the ten first days of the experiment were crucial for its further growth. 

To our knowledge, the life history of *T. confusum* strains infesting different grain commodities has not been researched yet. To fulfill this objective, we first examined the development, survival, longevity and fecundity of two laboratory strains from Greece and Serbia fed on cracked barley and cracked white rice and, second, we calculated several demographic parameters of these two strains to assess their potential population growth. 

## 2. Materials and Methods

### 2.1. Insect Strains

The strains were provided by the Laboratory of Agricultural Zoology and Entomology of the Agricultural University of Athens, Greece and by the Institute of Pesticides and Environmental Protection, Belgrade, Serbia. The Greek strain had been reared for more than 17 years on wheat flour plus 5% brewer’s yeast, at 30 °C, 65% relative humidity and continuous darkness, while the Serbian strain had been reared for more than 25 years on wheat flour plus 5% brewer’s yeast at 25 °C and 65% relative humidity and continuous darkness. The Serbian strain was transferred to the Athens Laboratory and kept under the same conditions as the Greek strain for one generation. The founding individuals of the Greek and Serbian strains were originally collected from Greek and Serbian storage facilities, respectively.

### 2.2. Commodities

Clean and free of infestation and pesticides hulless barley and white rice were used in the tests. Prior to experimentation, the moisture of the tested grains was adjusted to 13.5 ± 0.5% by heating them in an oven at 50 °C or by adding distilled water [53,54]. The moisture was measured by a calibrated moisture meter (mini GAC plus, Dickey–John Europe S.A.S., Colombes, France).

### 2.3. Development and Survival of Immatures

Both grains were cracked by an electric grinder Multi 600 (Izzy, Benroubi S.A., Amaroussion, Greece). Subsequently, they were sieved in a standard testing sieve having openings of 2.36 mm (Advantech Manufacturing Inc., New Berlin, WI, USA) and in one with 2.00 mm openings (Retsch GmbH, Haan, Germany) to remove dusts and obtain uniform particles of cracked grains. Taken from each strain, 100 unsexed approximately 7-day-old adults were transferred to two 250 mL-glass vials, each containing 125 g of fresh, pre-sieved hard wheat flour, and then placed into an incubator set at 30 °C, 65% relative humidity and continuous darkness for one day. Afterwards, flour from each vial was sieved to remove adult individuals and laid eggs, using standard testing sieves with 0.85 and 0.25 mm openings (Advantech Manufacturing Inc., New Berlin, WI, USA). The eggs found on the mesh of the sieve with 0.25 mm openings were gently and separately transferred to petri dishes (5.5 cm diameter, 1 cm height) with a fine brush (Cotman 111 No 000, Winsor and Newton, London, UK) that did not contain food. The lids of the dishes had a central circular opening (1.5 cm diameter) which were covered by muslin gauze allowing adequate ventilation inside the dishes. Then, dishes were placed inside incubators set at 30 °C, 65% relative humidity and continuous darkness and inspected every 24 h for duration and survival under a SZX9 Olympus stereomicroscope with 57× total magnification (Bacacos S.A., Athens, Greece). Regarding each strain and commodity, the experiment was initiated with a cohort of 100 eggs. Using a fine brush (Cotman 111 No 000, Winsor and Newton, London, UK), newly hatched *T. confusum* larvae were very carefully placed separately inside dishes (5.5 cm diameter, 1 cm height) that contained 1 g cracked barley or cracked white rice. All quantities of 1 g were weighed with a Precisa XB3200D compact balance (Alpha Analytical Instruments, Gerakas, Greece). The dishes were placed into incubators set at 30 °C, 65% relative humidity and continuous darkness for the entire experimental period. The duration and survival of larval and pupal stages were inspected every 24 h under the aforementioned stereomicroscope. All larvae of both strains died in cracked white rice before reaching the pupal stage, therefore the experiment was continued with strains on cracked barley.

### 2.4. Adult Longevity and Reproductive Capacity

One-day-old pupae were sexed following Halstead [55]. Each newly emerged pair (a male and a female) was placed in a different dish (5.5 cm diameter, 1 cm height) that contained 1 g of cracked barley then placed into incubators set at 30 °C, 65% relative humidity and continuous darkness. Adult longevity and female fecundity were inspected daily until death of the male and female. Finally, the progeny sex ratio was calculated for each strain on the basis of 100 randomly selected pupae originating from eggs obtained from the coupled females.

### 2.5. Statistical Analysis

The data on the larval and pupal development, female and male longevity, as well as on the female fecundity were examined via the Shapiro–Wilk normality test which indicated departure from a normal distribution. Therefore, all pairwise comparisons were done using the Mann–Whitney Rank Sum Test. The Kaplan–Meier method was used to estimate the survival curves of the two strains fed on cracked barley and cracked white rice. Additionally, the Kaplan–Meier estimate was used to derive the mean survival times and their 95% confidence intervals (C.I.). All analyses were done using the statistical package SigmaPlot 14.0 [56].

Concerning each *T. confusum* strain fed on cracked barley, the following demographic parameters were calculated [36]: the net reproductive rate $R_{0}=\sum \left(l_{x}\times m_{x}\right)$ , i.e., the per capita rate of progeny production in an interval equal to cohort study interval (*l_x_* corresponds to the cohort survival to age *x* and *m_x_* the age specific fecundity); the intrinsic rate of increase (*r_m_*) $\sum \left(e^{r_{m}\times x}\times l_{x}\times m_{x}\right)=1$ , i.e., the rate of natural increase in a closed population (that is subjected to constant age-specific schedules of fertility and mortality for a long period); the finite rate of increase $\lambda =e^{r_{m}}$, i.e., the rate at which the population will increase in each time step, the mean generation time $T=\frac{\ln R_{0}}{r_{m}}$, i.e., the time required for the population to increase by a factor equal to the net reproductive rate and the doubling time $DT=\frac{\ln2}{r_{m}}$, i.e., the time required for the population to double. Significant differences between demographic parameters were tested via the superposition of 95% C.I. (Wald test), which were obtained by bootstrapping in R [57]. Particularly, for each treatment we sampled ten thousand individuals to obtain the 95% confidence intervals. When *T. confusum* fed on cracked white rice, no demographic analysis was conducted as both strains did not complete their development.

## 3. Results

The biological features of the *T. confusum* strains fed on cracked barley are presented in Table 1. Larval development was significantly shorter for the Serbian compared to the Greek strain (37.7 and 49.7 days, respectively), but pupal development did not differ between the two strains (6.2 days for both strains). Additionally, male longevity did not differ between the Greek and Serbian strains (144.4 and 151.4 days, respectively). While female longevity was significantly shorter for the Serbian (151.7 days) compared to the Greek strain (186.6 days), fecundity did not differ between the two strains (11.3 and 17.7 eggs/female for the Greek and Serbian strain, respectively). When both *T. confusum* strains fed on cracked white rice they did not complete their development.

**Figure 1:**
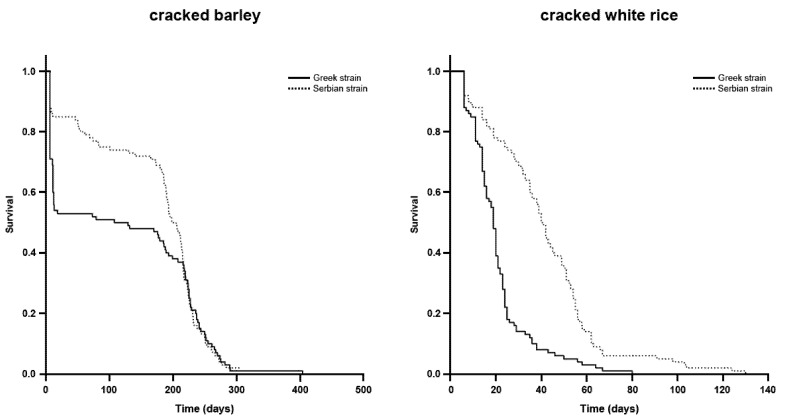
Survival curves of two *Tribolium confusum* strains fed on cracked barley and cracked white rice.

The survival curves for the Serbian and Greek strains on both of the tested commodities are presented in Figure 1. Mean survival times were 120.8 and 21.1 days for the Greek strain when fed on cracked barley and cracked white rice, respectively, where the corresponding values were 169.9 and 41.4 days for the Serbian strain (Table 2). Based on the 95% C.I. criterion, mean survival times were longer for the Serbian strain.

The values of the net reproductive rate, the intrinsic rate of increase and the finite rate of increase on cracked barley were significantly higher for the Serbian (7.27 females/female, 0.025 female/female/day and 1.026, respectively) compared to the Greek strain (2.91 females/female, 0.014 females/female/day and 1.014, respectively) (Table 3). The mean generation time, as well as the doubling time did not differ between the two strains (76.8 and 78.1 days, 41.2 and 27.7 days, for the Greek and Serbian strains, respectively).

## 4. Discussion

Our study reveals several clear findings on the performance of *T. confusum*. This species did not complete the development on cracked white rice in contrast to cracked barley. Previous reports have documented that diet affects several biological features of *T. confusum*. Adult emergence ranged from 3.8 to 62.4 individuals on various commercial types of milk powder within a period of 65 days [58]. A top patent flour enriched with bran and germ enhanced fecundity of *T. confusum* (10.0 eggs/day/female) compared to patent flours enriched with bran only (6.3 eggs/day/female), germ only (9.1 eggs/day/female), vitamins in different combinations (3.3–7.2 eggs/day/female), non-enriched patent flour (3.2 eggs/day/female) or whole wheat flour (7.0 eggs/day/female) [59]. Among several types of diets, wheat flour containing 5% (w/w) brewer’s yeast provided the shortest interval (17 days) at which *T. confusum* larvae became pupae and led to heavier pupae (3 mg) [60]. Similarly, Prus et al. [61] reported that the absence of yeast from wheat flour resulted to reduced fecundity and reproductive capacity of *T. confusum*. By testing different combinations of cracked wheat kernels, wheat starch and crude α-amylase inhibitor, Warchalewski et al. [62] altered the larva-to-adult *T. confusum* period, ranging from 22.1 to 46.3 days, and the percentage of adult emergence, ranging from 56.0 to 76.0%. Grain commodities, e.g., spikelets of emmer and spelt, exhibited different levels of susceptibility to *T. confusum* infestations with the former favoring its population growth more than the latter [63]. However, non-grain commodities, such as soybean and pigeon pea flours, reduced fecundity, extended the larval or pupal developmental period and reduced the survival of adults compared to wheat flour [64]. Furthermore, they are able to cause variable levels of weight losses to different varieties of stored-maize ranging from 2.56 to 21.63 g maize/100 g maize six months post-treatment [65].

Our study also provides evidence that different strains of *T. confusum* may exhibit variable biological and demographic performances. Although this species did not complete development when fed on cracked white rice, the Serbian strain showed a higher survival than the Greek strain. Moreover, larvae of the Serbian strain exhibited faster larval development on cracked barley. Additionally, the survival of the Serbian strain was higher on this commodity. The intrinsic rate of increase is an indicator of the potential growth of an insect population [34,66,67]. As this value is higher for the Serbian strain, we expect to increase its population faster compared to the Greek strain. This fact also is depicted on the values of the finite rate of increase and the net reproductive value which are also higher for the Serbian strain. However, the mean generation time did not differ between the two strains, which may appear somewhat surprising. This parameter represents the average time for a population to increase by a factor equal to the net reproductive rate [68]. Therefore, although the Serbian strain has a shorter larval development, a longer adult time period may be needed to reach its higher net reproductive rate compared to the Greek strain.

The fact that both tested strains did not complete development on cracked white rice proves that this commodity is unsuitable for *T. confusum*. This species can only spend a short time period on cracked white rice, as this commodity can act only as a temporary host for it. This issue could be attributed to the nutritional value of this commodity. A portion of its nutrient ingredients such as protein, fat, vitamins and minerals are lost during the milling process, which includes the removal of bran and germ from the kernels [69,70]. Development and fecundity of *T. confusum* are increased when thiamine, riboflavin and niacin exist in its diet [59]. However, these vitamins are substantially reduced during processing of rice [70]. Particularly, in white rice, the amount of riboflavin ranges between 0.2 and 0.6 μg/g [69] which is lower than the minimum required level for the development of *T. confusum* larvae (1–2 μg/g) [59,71]. To contrast, both strains completed their cycle on barley that contained riboflavin ranging between 1.5 and 2.85 μg/g [72,73]. Thus, white rice should be considered as an unsuitable commodity for *T. confusum*. However, on the basis of our results, white rice can host larvae of both strains for a considerable period of time before they die, i.e., maximally 124 and 61 days for the Serbian and Greek strains, respectively. During these intervals, larvae may be moved through the transfer of commodities between or among storage facilities and through their standard cleaning [74,75,76]. Found in the new storage environment and/or location in the same storage facility, more suitable grain commodities than white rice may exist for *T. confusum* larval development (e.g., barley, as our study indicates). Considering that stored-product insects, including *T. confusum*, are attracted variably by the volatile odors of stored-grains [7], new infestations may be initiated. Therefore, white rice could be considered as a vehicle of temporal survival of different *T. confusum* strains. Whether larvae that have their feeding commodity altered from white rice to a different one would be able to pupate and provide fecund adults merits further experimentation. The discovery of grain or non-grain commodities which marginally allow the development of stored-product insects is important since it illuminates the potential paths they follow for survival and further expansion [54,77]. 

Based on the values of the demographic parameters, both strains are able to increase their population on cracked barley. Therefore, this commodity can be suitable for *T. confusum*. One of the most important findings of the current study is that, although both strains successfully completed their cycle on barley, the females of the Serbian strain exhibited a higher net reproductive rate and faster larvae development. Despite the fact that our tests were conducted under the same abiotic and biotic conditions, the Serbian strain had been adapted for >25 years at 25 °C, although we reared it at 30 °C for one generation, while the Greek strain had been adapted for >17 years at 30 °C. This issue might partially explain the obtained differences between the two strains, taking into account that alteration of temperature and relative humidity led to alteration of the relative rate of increase of various lineages of another closely related stored-product pest *T. castaneum* [78]. Moreover, Kavallieratos et al. [25] found that the increase of temperature increased the food uptake of seven European strains of *T. confusum* differently. This fact was interpreted as a variable susceptible to natural insecticides, i.e., diatomaceous earths (DEs) as wheat protectants. Consequently, the increase in temperature may have activated the Serbian strain to become more fecund. Although the tested strains originated from neighboring countries (Greece and Serbia), they performed differently on barley on the basis of the calculated values of their life history parameters. Similarly, Vayias et al. [79] found significant differences in the mortality levels of adults between a Danish strain and a German strain of *T. confusum* seven days post-exposure on wheat treated with DEs. Later, Athanassiou et al. [26] showed that spinosad killed significantly more adults and larvae of a *T. confusum* strain which originated from Italy compared to a strain from Greece on wheat after 14 and 21 days of exposure. To contrast, according to Wade [29], two wild strains of *T. confusum* from the U.S.A. and Spain did not exhibit significant differences in the mean rates of increase of their populations over 14 generations on wheat flour. 

## 5. Conclusions

Our study shows that different *T. confusum* strains may exhibit significant differences in their biological characteristics and demographic traits, with regard to the feeding commodity. However, it should be noted that the obtained differences also could be due to various causes such as rearing abiotic (e.g., temperature) or biotic (e.g., wheat varieties that form flour) conditions. Therefore, different strains may alter the potential growth and spread of a population, as well as the level of infestation of stored products. Taking a practical point of view, the knowledge of the life histories of certain strains leads to the optimization of their cultures in the insectary for the production of adequate numbers of individuals that are necessary for laboratory tests. Since origin impacts the response of *T. confusum* strains to insecticides either as grain protectants or as fumigants, or even as topical applications [25,79,80,81,82,83], and taking into account the results of the current study, further research is needed to shed light on the life history of such strains infesting different types of commodities when they are treated with insecticides. The fact that different *T. confusum* strains show remarkable differences in their population dynamics when infesting certain suitable grain commodities should not be overlooked because it may lead to considerable losses. The study of their life history parameters on different abiotic scenarios would be a tool that enables the estimation of their temporal population fluctuation, triggering accurate management treatments against *T. confusum*.

## Figures and Tables

**Table 1:** Duration of immature development, female and male longevity in days (mean ± SE, median) and female fecundity (eggs/female) of two strains of *Tribolium confusum* (mean, 95% C.I.) fed on cracked barley. Medians within a column followed by the same letter are not statistically different (Mann–Whitney Rank Sum Test at *a* = 0.05).

Strain	Larva	Sample Size	Pupa	Sample Size	Female	Sample Size	Male	Sample Size	Fecundity	Sample Size
Greek	49.7 ± 2.2	53	6.2 ± 0.1	53	186.6 ± 14.9	18	144.4 ± 9.9	35	11.3 ± 2.7	18
	43.0 a		6.0 a		185.5 a		165.5 a		9.0 a	
Serbian	37.7 ± 0.8	85	6.2 ± 0.1	83	151.7 ± 9.0	43	151.4 ± 9.9	40	17.7 ± 3.0	43
	37.0 b		6.0 a		166.0 b		159.5 a		12.0 a	
*U*	1086.500		2006.000		249.500		684.000		331.5	
*p*	<0.001		0.290		0.030		0.869		0.381	

**Table 2:** Survival times (mean ± SE) in days of two strains of *Tribolium confusum* fed on cracked barley and cracked white rice. Cracked barley means followed by different uppercase letter are statistically different (95% C.I.). Cracked white rice means followed by different lowercase letter are statistically different (95% C.I.).

Strain	Commodity	Mean	95% C.I.
Greek	Cracked barley	120.8 ± 11.7 A	98.5–143.1
Serbian	Cracked barley	169.9 ± 9.0 B	152.2–187.5
Greek	Cracked white rice	21.1 ± 1.4 a	18.4–23.7
Serbian	Cracked white rice	41.4 ± 2.5 b	36.6–46.3

**Table 3:** Demographic parameters of two strains of *Tribolium confusum* (mean, 95% C.I.) fed on cracked barley.

Strain	Net Reproductive Rate (Females/Female) $R_{0}=\sum \left(l_{x}\times m_{x}\right)$		Intrinsic Rate of Increase (Females/Female/Day) $\sum \left(e^{r_{m}\times x}\times l_{x}\times m_{x}\right)=1$		Finite Rate of Increase $\lambda =e^{r_{m}}$		Mean Generation Time (Days) $T=\frac{\ln R_{0}}{r_{m}}$		Doubling Time (Days) $DT=\frac{\ln2}{r_{m}}$	
	Mean	95% C.I.	Mean	95% C.I.	Mean	95% C.I.	Mean	95% C.I.	mean	95% C.I.
Greek	2.91	1.62–4.41	0.014	0.006–0.020	1.014	1.006–1.020	76.8	69.4–90.3	41.2	34.4–117.5
Serbian	7.27	5.14–10.01	0.025	0.020–0.030	1.026	1.020–1.031	78.1	72.4–85.6	27.7	22.8–34.7

## References

[1] Pedersen J.R., Sauer D.B. (1992). Insects: Identification, damage, and detection. Storage of Cereal Grains and Their Products.

[2] Verheggen F., Ryne C., Olsson P.O.C., Arnaud L., Lognay G., Högberg H.E., Persson D., Haubruge E., Löfstedt C. (1992). Electrophysiological and behavioral activity of secondary metabolites in the confused flour beetle, *Tribolium confusum*. J. Chem. Ecol..

[3] Mason L.J., McDonough M., Hagstrum D.W., Phillips T.W., Cuperus G. (2012). Biology, behavior, and ecology of stored grain and legume insects. Stored Product Protection.

[4] Rees D. (2004). Insects of Stored Products.

[5] Kumar R. (2017). Insect Pests on Stored Grain. Biology, Behavior, and Management Strategies.

[6] Storey C.L., Watson S.A., Ramstad P.E. (1987). Effect and control of insects affecting corn quality. Corn Chemistry and Technology.

[7] Trematerra P., Sciarreta A., Tamasi E. (2000). Behavioural responses of *Oryzaephilus surinamensis*, *Tribolium castaneum* and *Tribolium confusum* to naturally and artificially damaged *durum* wheat kernels. Entomol. Exp. Appl..

[8] Hagstrum D.W., Subramanyam B. (2009). Stored-Product Insect Resource.

[9] Robinson W.H. (2005). Urban Insects and Arachnids.

[10] Hill D.S. (2003). Pests of Storage Foodstuffs and Their Control.

[11] Mahroof R.M., Hagstrum D.W., Hagstrum D.W., Phillips T.W., Cuperus G. (2012). Biology, behavior, and ecology of insects in processed commodities. Stored Product Protection.

[12] Kavallieratos N.G., Athanassiou C.G., Hatzikonstantinou A.N., Kavallieratou H.N. (2011). Abiotic and biotic factors affect efficacy of chlorfenapyr for control of stored-product insect pests. J. Food Prot..

[13] Kavallieratos N.G., Athanassiou C.G., Boukouvala M.C. (2013). Insecticidal effect of chlorantraniliprole against major stored product insect pests in different grain commodities under laboratory tests. Pest Manag. Sci..

[14] Athanassiou C.G., Kavallieratos N.G. (2014). Evaluation of spinetoram and spinosad for control of *Prostephanus truncatus, Rhyzopertha dominica*, *Sitophilus oryzae* and *Tribolium confusum* on stored grains under laboratory tests. J. Pest Sci..

[15] Kavallieratos N.G., Athanassiou C.G., Korunic Z., Mikeli N.H. (2015). Evaluation of three novel diatomaceous earths against three stored-grain beetle species on wheat and maize. Crop Prot..

[16] Mullen G.R., Durden L.A. (2019). Medical and Veterinary Entomology.

[17] Kljajić P., Perić P. (2007). Altered susceptibility of granary weevil *Sitophilus granarius* (L.) (Coleoptera: Curculionidae) populations to insecticides after selection with pirimiphos-methyl and deltamethrin. J. Stored Prod. Res..

[18] Clark L.R., Geier P.W., Hughes R.D., Morris R.F. (1967). The Ecology of Insect Populations in Theory and Practice.

[19] Stillwell R.C., Fox C.W. (2009). Geographic variation in body size, sexual size dimorphism and fitness components of a seed beetle: Local adaptation versus phenotypic plasticity. Oikos.

[20] Gerken A.R., Scully E.D., Campbell J.F. (2018). Red flour beetle (Coleoptera: Tenebrionidae) response to volatile cues varies with strain and behavioral assay. Environ. Entomol..

[21] Romano D., Kavallieratos N.G., Athanassiou C.G., Stefanini C., Canale A., Benelli G. (2016). Impact of geographical origin and rearing medium on mating success and lateralization in the rice weevil, *Sitophilus oryzae* (L.) (Coleoptera: Curculionidae). J. Stored Prod. Res..

[22] Arnaud L., Brostaux Y., Lallemand S., Haubruge E. (2005). Reproductive strategies of *Tribolium* flour beetles. J. Insect Sci..

[23] Rigaux M., Haubruge E., Fields P.G. (2001). Mechanisms for tolerance to diatomaceous earth between strains of *Tribolium castaneum* (Coleoptera: Tenebrionidae). Entomol. Exp. Appl..

[24] Kljajić P., Perić I. (2006). Susceptibility to contact insecticides of granary weevil *Sitophilus granarius* (L.) (Coleoptera: Curculionidae) originating from different locations in the former Yugoslavia. J. Stored Prod. Res..

[25] Kavallieratos N.G., Athanassiou C.G., Vayias B.J., Maistrou S.N. (2007). Influence of temperature on susceptibility of *Tribolium confusum* (Coleoptera: Tenebrionidae) populations to three modified diatomaceous earth formulations. Fla Entomol..

[26] Athanassiou C.G., Kavallieratos N.G., Chintzoglou G.J. (2008). Effectiveness of spinosad dust against different European populations of the confused flour beetle, *Tribolium confusum* Jacquelin du Val. J. Stored Prod. Res..

[27] Giga D.P., Smith R.H. (1983). Comparative life history studies of four *Callosobruchus* species infesting cowpeas with special reference to *Callosobruchus rhodesianus* (Pic) (Coleoptera: Bruchidae). J. Stored Prod. Res..

[28] Collins P.J., Mulder J.C., Wilson D. (1989). Variation in life history parameters of *Oryzaephilus surinamensis* (L.). (Coleoptera: Silvanidae). J. Stored Prod. Res..

[29] Wade M.J. (1991). Genetic variance for rate of population increase in natural populations of flour beetles, *Tribolium* spp.. Evolution.

[30] Papanikolaou N.E., Milonas P.G., Kontodimas D.C., Demiris N., Matsinos Y.G. (2013). Temperature-dependent development, survival, longevity, and fecundity of *Propylea quatuordecimpunctata* (Coleoptera: Coccinellidae). Ann. Entomol. Soc. Am..

[31] Papachristos D.P., Katsarou I., Michaelakis A., Papanikolaou N.E. (2015). Influence of different species of aphid prey on the immature survival and development of four species of aphidophagous coccinellids (Coleoptera: Coccinellidae). Eur. J. Entomol..

[32] Skourti A., Kavallieratos N.G., Papanikolaou N.E. (2019). Laboratory evaluation of development and survival of *Tribolium castaneum* (Herbst) (Coleoptera: Tenebrionidae) under constant temperatures. J. Stored Prod. Res..

[33] Kavallieratos N.G., Karagianni E.S., Papanikolaou N.E. (2019). Life history of *Trogoderma granarium* Everts (Coleoptera: Dermestidae) on peeled barley, peeled oats and triticale. J. Stored Prod. Res..

[34] Papanikolaou N.E., Milonas P.G., Kontodimas D.C., Demiris N., Matsinos Y.G. (2014). Life table analysis of *Propylea quatuordecimpunctata* (Coleoptera: Coccinellidae) at constant temperatures. Ann. Entomol. Soc. Am..

[35] Papanikolaou N.E., Kavallieratos N.G., Kondakis N., Boukouvala M.C., Nika E.P., Demiris N. (2019). Elucidating fitness components of the invasive dermestid beetle *Trogoderma granarium* Everts (Coleoptera: Dermestidae) at constant temperatures, combining deterministic and stochastic demography. PLoS ONE.

[36] Carey J.R. (1993). Applied Demography for Biologists with Special Emphasis on Insects.

[37] Maia A.H.N., Luiz A.J.B., Campanhola C. (2000). Statistical inference on associated fertility life tables parameters using jackknife technique: Computational aspects. J. Econ. Entomol..

[38] Howe R.W. (1966). Developmental period, and the shape of the curve representing it in stored products beetles. J. Stored Prod. Res..

[39] Hagstrum D.W., Throne A.E. (1989). Predictability of stored-wheat insect population trends from life history traits. Environ. Entomol..

[40] Hagstrum D.W., Milliken G.A. (1991). Modeling differences in insect developmental times between constant and fluctuating temperatures. Ann. Entomol. Soc. Am..

[41] Hagstrum D.W., Milliken G.A. (1988). Quantitative analysis of temperature, moisture, and diet factors affecting insect development. Ann. Entomol. Soc. Am..

[42] Hagstrum D.W., Subramanyam B. (2006). Fundamentals of Stored-Product Entomology.

[43] Boina D.R., Subramanyam B., Alavi S. (2008). Dynamic model for predicting survival of mature larvae of *Tribolium confusum* during heat treatments. J. Econ. Entomol..

[44] Mahroof R.M., Phillips T.W. (2008). Life history parameters of *Lasioderma serricorne* (F.) as influenced by food sources. J. Stored Prod. Res..

[45] Golizadeh A., Abedi Z. (2016). Comparative performance of the khapra beetle, *Trogoderma granarium* Everts (Coleoptera: Dermestidae) on various wheat cultivars. J. Stored Prod. Res..

[46] Majd Marani S., Nouri Ganbalani G., Borzoui E. (2017). The effect of maize hybrid on biology and life table parameters of the *Trogoderma granarium* (Coleoptera: Dermestidae). J. Econ. Entomol..

[47] Predojević D.Z., Vukajlović F.N., Tanasković S.T., Gvozdenac S.M., Pešić S.B. (2017). Influence of maize kernel state and type on life history of *Plodia interpunctella* (Lepidoptera: Pyralidae). J. Stored Prod. Res..

[48] Karimi Pormehr M.S., Borzoui E., Naseri B., Dastjerdi H.R., Mansouri S.M. (2018). Two-sex life table analysis and digestive physiology of *Sitotroga cerealella* (Olivier) (Lepidoptera: Gelechiidae) on different barley cultivars. J. Stored Prod. Res..

[49] Nemati Kalkhoran M., Razmjou J., Borzoui E., Naseri B. (2018). Comparison of life table parameters and digestive physiology of *Rhyzopertha dominica* (Coleoptera: Bostrichidae) fed on various barley cultivars. J. Insect Sci..

[50] Hardman J.M. (1976). Life table data for use in deterministic and stochastic simulation models predicting the growth of insect populations under Malthusian conditions. Can. Entomol..

[51] Hardman J.M. (1976). Deterministic and stochastic models simulating the growth of insect populations over a range of temperatures under Malthusian conditions. Can. Entomol..

[52] Daly P.J., Ryan M.F. (1983). Density-related mortality of the flour beetle, *Tribolium confusum* Duval. Res. Popul. Ecol..

[53] Athanassiou C.G., Kavallieratos N.G., Boukouvala M.C., Nika E.P. (2017). Influence of commodity on the population growth of the larger grain borer, *Prostephanus truncatus* (Horn) (Coleoptera: Bostrychidae). J. Stored Prod. Res..

[54] Athanassiou C.G., Kavallieratos N.G., Brabec D.L., Agrafioti P., Sakka M., Campbell J.F. (2019). Using immobilization as a quick diagnostic indicator for resistance to phosphine. J. Stored Prod. Res..

[55] Halstead D.G. (1962). External sex differences in stored-products Coleoptera. Bull. Entomol. Res..

[56] Systat Software (2017). SigmaPlot for Windows Version 14.0.

[57] R Development Core Team R: A Language and Environment for Statistical Computing. R Foundation for Statistical Computing. http://www.Rproject.org.

[58] Yoshida T. (1975). Rearing twelve coleopterous species and one psocid infesting cereal products on milk powder. Food Hyg. Saf. Sci..

[59] Hämäläiner M., Loschiavo S.R. (1977). Effect of synthetic B-vitamin and natural enrichment of flour on larval development and fecundity of *Tribolium confusum* and *Tribolium castaneum*. Entomol. Exp. Appl..

[60] Loschiavo S.R., White N.D.G. (1986). Effects of diet and population density on larval development and pupal weight of *Tribolium confusum*. Can. Entomol..

[61] Prus T., Bijok P., Prus M. (2000). Reaction of phenotypes of *Tribolium castaneum* (Herbst) and *T. confusum* (Duval) to changes in their diet. Pol. J. Ecol..

[62] Warchalewski J.R., Gralik J., Winiecki Z., Nawrot J., Piasecka Kwiatkowska D. (2002). The effect of wheat α-amylase inhibitors incorporated into wheat-based artificial diets on development of *Sitophilus granarius* L., *Tribolium confusum* Duv., and *Ephestia kuehniella* Zell. J. Appl. Entomol..

[63] Gałęcki R., Bakuła T., Wojtacki M., Żuk Gołaszewska K. (2019). Susceptibility of ancient wheat species to storage pests *Sitophilus granarius* and *Tribolium confusum*. J. Stored Prod. Res..

[64] Ajayi E.O., Oladipuro S.O., Ajisafe O. (2019). Influence of processing and substrate variety on survival and development of *Tribolium confusum* (Coleoptera: Tenebrionidae). Arch. Phytopathol. Plant Prot..

[65] Kumari S., Memon N., Shah M.A., Mal B. (2017). Resistance of different maize varieties against flour beetles, *Tribolium castaneum* and *Tribolium confusum* (Coleoptera: Tenebrionidae). Pure Appl. Biol..

[66] Kontodimas D.C., Milonas P.G., Stathas G.J., Papanikolaou N.E., Skourti A., Matsinos Y.G. (2008). Life table parameters of the aphid predators *Coccinella septempunctata*, *Ceratomegilla undecimnotata* and *Propylea quatuordecimpunctata* (Coleoptera: Coccinellidae). Eur. J. Entomol..

[67] Zeki E., Papanikolaou N.E., Demiris N., Kontodimas D.C. (2015). Comparison of the demographic parameters and survival of two phenotypes of *Harmonia axyridis* (Coleoptera: Coccinellidae). Eur. J. Entomol..

[68] Borges I., Hemptinne J.L., Soares A.O. (2013). Contrasting population growth parameters of the aphidophagous *Scymnus nubilus* and the coccidophagous *Nephus reunioni*. Biocontrol.

[69] Krishna J.G., Chandrasekaran M., Chandrasekaran M. (2013). Cereals. Valorization of Food Processing by-Products.

[70] Saleh A.S.M., Wang P.W.N., Yang L., Xiao Z. (2019). Brown rice versus white rice: Nutritional quality, potential health benefits, development of food products, and preservation technologies. Compr. Rev. Food Sci. Food Saf..

[71] Fraenkel G., Blewett M. (1943). Vitamins of the B-group required by insects. Nature.

[72] Shewry P.R., Shewry P.R., Ulrich S.E. (2014). Minor components of the barley grain: Minerals, lipids, terpenoids, phenolics, and vitamins. Barley. Chemistry and Technology.

[73] Rosentrater K.A., Everts A.D. (2018). Kent’s Technology of Cereals. An Introduction for Students of Food Science and Agriculture.

[74] Arthur F.H. (2015). Food source and residual efficacy of chlorfenapyr as a surface treatment on sealed and unsealed concrete. J. Stored Prod. Res..

[75] Kavallieratos N.G., Boukouvala M. (2018). Efficacy of four insecticides on different types of storage bags for the management of *Trogoderma granarium* Everts (Coleoptera: Dermestidae) adults and larvae. J. Stored Prod. Res..

[76] Lazarević M., Kavallieratos N.G., Nika E.P., Boukouvala M.C., Skourti A., Žikić V., Papanikolaou N.E. (2019). Does the exposure of parental female adults of the invasive *Trogoderma granarium* Everts to pirimiphos-methyl on concrete affect the morphology of their adult progeny? A geometric morphometric approach. Environ. Sci. Pollut. Res..

[77] Kavallieratos N.G., Athanassiou C.G., Boukouvala M.C., Tsekos G.T. (2019). Influence of different non-grain commodities on the population growth of *Trogoderma granarium* Everts (Coleoptera: Dermestidae). J. Stored Prod. Res..

[78] Wade M.J. (1990). Genotype-environment interaction for climate and competition in a natural population of flour beetles, *Tribolium castaneum*. Evolution.

[79] Vayias B.J., Athanassiou C.G., Kavallieratos N.G., Buchelos C.T. (2006). Susceptibility of different European populations of *Tribolium confusum* (Coleoptera: Tenebrionidae) to five diatomaceous earth formulations. J. Econ. Entomol..

[80] Zettler J.L., Arthur F.H. (1997). Dose-response tests on red flour beetle and confused flour beetle (Coleoptera: Tenebrionidae) collected from flour mills in the United States. J. Econ. Entomol..

[81] Rossi E., Cosimi S., Loni A. (2010). Insecticide resistance in Italian populations of *Tribolium* flour beetles. Bull. Insectol..

[82] Aulicky R., Stejskal V., Dlouhy M., Liskova J. (2015). Validation of hydrogen cyanamide fumigation in flourmills to control the confused flour beetle. Chech J. Food Sci..

[83] Athanassiou C.G., Kavallieratos N.G., Boukouvala M.C. (2016). Population growth of the khapra beetle, *Trogoderma granarium* Everts (Coleoptera: Dermestidae) on different commodities. J. Stored Prod. Res..

